# Broad metabolic sensitivity profiling of a prototrophic yeast deletion collection

**DOI:** 10.1186/gb-2014-15-4-r64

**Published:** 2014-04-10

**Authors:** Benjamin VanderSluis, David C Hess, Colin Pesyna, Elias W Krumholz, Tahin Syed, Balázs Szappanos, Corey Nislow, Balázs Papp, Olga G Troyanskaya, Chad L Myers, Amy A Caudy

**Affiliations:** 1 Department of Computer Science and Engineering, University of Minnesota Twin Cities, 200 Union St SE, Minneapolis, MN 55455, USA; 2 Department of Biology, Santa Clara University, 500 El Camino Real, Santa Clara, CA 95053, USA; 3 Department of Plant Biology, University of Minnesota Twin Cities, 1445 Gortner Avenue, Saint Paul, MN 55108, USA; 4 Institute of Biochemistry, Biological Research Centre, Hungarian Academy of Sciences, H-6701, Szeged, Hungary; 5 University of British Columbia, Pharmaceutical Sciences, 2405 Wesbrook Mall, Vancouver, BC V6T 1Z3, Canada; 6 Department of Computer Science, Princeton University, Princeton, NJ 08540, USA; 7 Lewis-Sigler Institute for Integrative Genomics, Princeton University, Princeton, NJ 08544, USA; 8 Donnelly Centre for Cellular and Biomolecular Research and Department of Molecular Genetics, University of Toronto, 160 College Street, Toronto, ON M5S 3E1, Canada

## Abstract

**Background:**

Genome-wide sensitivity screens in yeast have been immensely popular following the construction of a collection of deletion mutants of non-essential genes. However, the auxotrophic markers in this collection preclude experiments on minimal growth medium, one of the most informative metabolic environments. Here we present quantitative growth analysis for mutants in all 4,772 non-essential genes from our prototrophic deletion collection across a large set of metabolic conditions.

**Results:**

The complete collection was grown in environments consisting of one of four possible carbon sources paired with one of seven nitrogen sources, for a total of 28 different well-defined metabolic environments. The relative contributions to mutants' fitness of each carbon and nitrogen source were determined using multivariate statistical methods. The mutant profiling recovered known and novel genes specific to the processing of nutrients and accurately predicted functional relationships, especially for metabolic functions. A benchmark of genome-scale metabolic network modeling is also given to demonstrate the level of agreement between current *in silico* predictions and hitherto unavailable experimental data.

**Conclusions:**

These data address a fundamental deficiency in our understanding of the model eukaryote *Saccharomyces cerevisiae* and its response to the most basic of environments. While choice of carbon source has the greatest impact on cell growth, specific effects due to nitrogen source and interactions between the nutrients are frequent. We demonstrate utility in characterizing genes of unknown function and illustrate how these data can be integrated with other whole-genome screens to interpret similarities between seemingly diverse perturbation types.

## Background

Large scale gene deletion screens have become common in *Saccharomyces cerevisiae* due to efforts in the yeast community to assemble a near complete collection of non-essential single-mutant strains [1]. The subsequent refinement of mating-based high-throughput strain construction techniques such as synthetic genetic array (SGA) analysis [2] has further driven the creation of customized yeast deletion arrays. While quantitative single mutant fitness assays have been performed [3], they are generally limited to a single growth medium. A few notable exceptions have begun to explore this space [4-7], but the conditions of interest are often chosen with human therapeutic ends in mind and are limited to known drugs or small molecules of unknown biological effect. A decade and a half after the sequencing of the best-studied eukaryote, a systematic exploration of mutant growth across basic nutrient environments is conspicuously absent. These data would be valuable for metabolic researchers and computational biologists that attempt to model the metabolic network of the cell using methodologies such as flux balance analysis (FBA) [8] because the defined growth conditions are amenable to modeling.

Yeast strain collections used in previous high-throughput assays (that is, the deletion collection) are auxotrophic [1], and therefore unable to survive in minimal media unless provided additional nutrients. This requirement reflects the historical use of auxotrophic markers for genetic selection. The resulting requirement for nutrient supplementation precludes systematic testing of the yeast deletion collection on specific combinations of carbon and nitrogen sources because the auxotrophic nutrient supplements can also be used as carbon and nitrogen sources. Previous work has shown not only that nutrient supplementation can have different physiological consequences from genetic complementation [9] but also that auxotrophies can alter the expression of many other genes [10].

**Figure 1:**
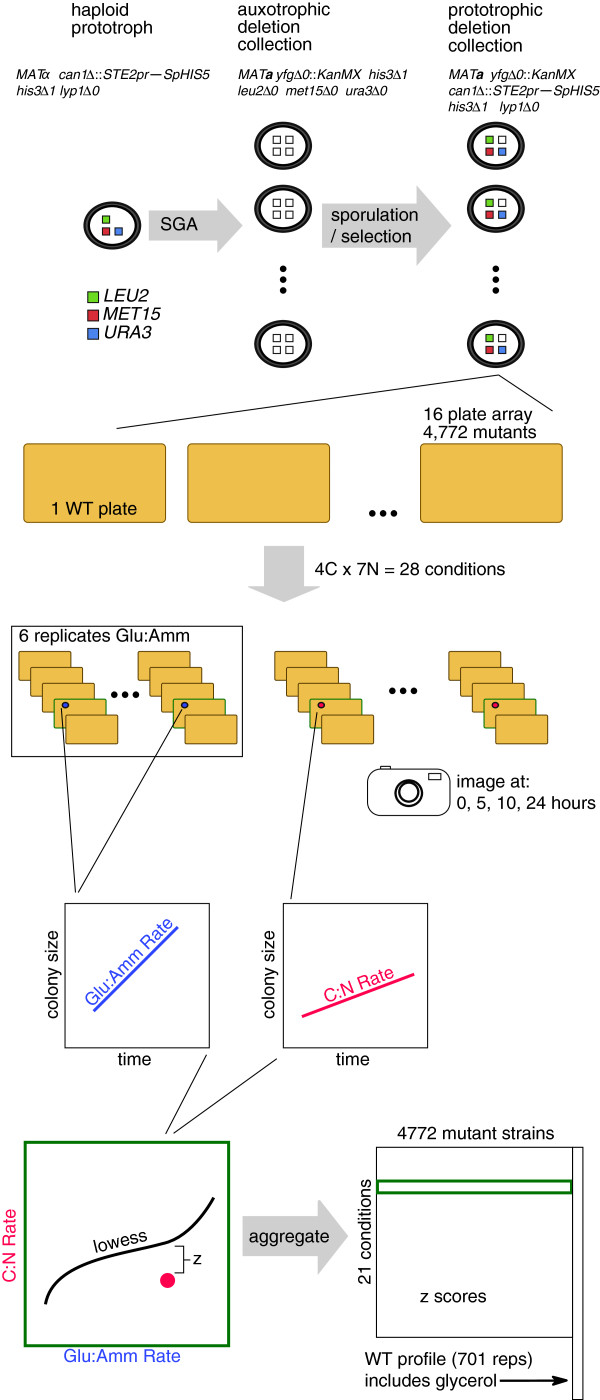
**Experimental overview.** A custom prototrophic strain is mated to the entire deletion collection and haploids are selected via SGA. The resulting prototrophic deletion collection is plated out onto 28 distinct metabolic media, and time course growth rate data are extracted from plate images. Growth rates are normalized to a glucose:ammonia reference (constructed from six replicates) and z-scores are calculated for each deletion, in each condition (except glycerol). WT, wild-type.

To address this deficiency in genome-scale data on growth in other, defined media, we constructed a prototrophic version of the yeast deletion collection and then screened this collection of 4,772 mutants against 28 defined minimal media conditions. These 28 conditions were formed by using all pairwise combinations of four carbon sources and seven nitrogen sources (Table 1, Figure 1). These screens of the prototrophic collection revealed numerous interactions between carbon and nitrogen sources with respect to wild-type growth rate, underscoring the need to perform growth experiments in a combinatorial fashion. Mutant data revealed condition-specific sensitivities across all conditions, including many effects for uncharacterized genes and mutants that are healthy under standard laboratory conditions. We show that the data have power to predict functional relationships between genes and are otherwise validated via a separate liquid assay as well as through comparison with previous studies involving galactose. We also present a method for distinguishing carbon and nitrogen effects from their combined profiles and additionally provide a benchmark of current constraint-based modeling techniques and their ability to predict our experimental data.

**Table 1:** Summary of conditions and hits called in each condition

**Fast/slow**	**Ammonium**	**Proline**	**Glutamate**	**Glutamine**	**Arginine**	**Urea**	**Allantoin**
Glucose	41.5/41*	186/417	133/354	169/286	173/920	135/219	95/284
Galactose	132/461	276/658	400/906	270/877	452/530	154/216	124/545
Ribose	312/345	981/462	306/412	291/192	437/46	388/345	379/492
Glycerol	NA	NA	NA	NA	NA	NA	NA

* Mean of six replicates. Note that this reflects the average number of false positives in the screen since glucose ammonium was the reference condition. NA, Noise from extremely slow growth on glycerol precluded identification of significant individual mutant effects.

## Results and discussion

### Prototrophic deletion set construction and profiling

Briefly, a *MATα* strain carrying the SGA marker [11,12] was crossed to the *MAT****a*** yeast deletion set [1], selected for diploids, and sporulated. Prototrophic haploids were selected using the SGA approach [11]. The final genotype of these 4,772 strains is *MAT****a****yfgΔ0::KanMX can1Δ::STE2pr-SpHIS5 his3Δ1 lyp1Δ0*. These strains were then pinned out onto plates containing one of four different carbon sources along with one of seven nitrogen sources. All 28 carbon:nitrogen combinations were included to produce a broad set of well-defined metabolic conditions. The plates were imaged in time course in order to estimate growth rates from measurements of colony size (Figure 1; see Materials and methods for details; Additional file 1).

### Yeast wild-type growth suggests carbon/nitrogen interactions

**Figure 2:**
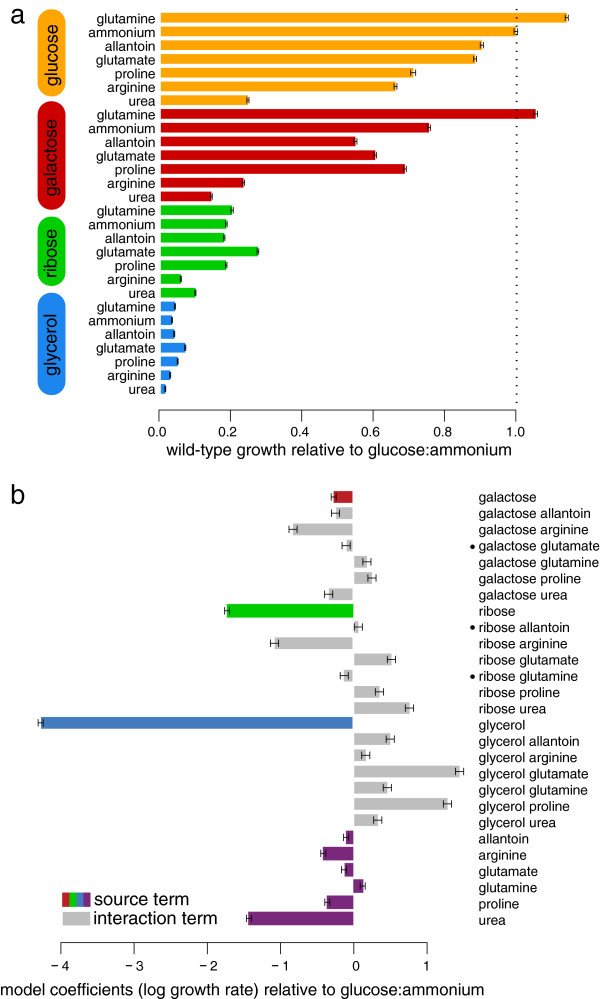
**Wild-type growth data in all conditions. (a)** Average wild-type growth rates in all conditions. Conditions are grouped and colored by carbon source. Nitrogen sources are ordered by growth rate when paired with glucose, and all values are relative to the glucose:ammonium rate. Error bars represent standard error from 701 wild-type replicates. **(b)** A linear model fit to log-transformed growth values. Terms for individual carbon and nitrogen sources are colored, interaction terms are gray. All but the three terms marked with a black circle are significant (*P* < 0.01); error bars represent standard error.

The mean growth rate of all wild-type replicates was calculated in each condition, which revealed extensive variation across the profiled conditions (Figure 2a; Additional file 2; Materials and methods). As expected, wild-type yeast grow substantially faster on glucose or galactose than on glycerol or ribose. Similarly, urea is a consistently poor nitrogen source with glutamine and ammonium generally preferred. To systematically examine the interactions between carbon and nitrogen sources over our entire dataset, a linear model was fit to the logarithm of wild-type growth rates under the assumption that independent contributions to growth rate would combine multiplicatively (a multiplicative model fit better than simple alternatives such as an additive formulation). Indeed, the model suggests that pairs of nitrogen and carbon sources commonly interact to produce a wild-type growth rate phenotype that is different from what might be predicted assuming independent contributions, evidenced by the fact that the majority of the interaction terms in the linear model were significant (Figure 2b). For example, consider the apparent increase in growth rate observed under ribose:glutamate when compared to glucose:glutamate (Figure 2a), observable as a positive interaction between ribose and glutamate (Figure 2b). When paired with glucose, glutamate is the nitrogen source that yields the fourth fastest growth rate. However, when paired with a much poorer carbon source (for example, ribose or glycerol) glutamate becomes the nitrogen source that yields the fastest growth rate. This interaction is likely caused by the ability of the cell to utilize glutamate not only as a source of nitrogen, but as a secondary carbon source in the presence of a poor primary carbon source. When glutamate is deaminated for use as a nitrogen source, alpha-ketoglutarate is produced and can be subsequently utilized for energy production via the tricarboxylic acid cycle. This dual role is not specific to glutamate. For example, glutamine is utilized in a similar manner, though the ratio of 'free' carbon skeletons per nitrogen produced is less efficient (1:2 as opposed to 1:1). Despite the fact that many of the nitrogen sources share this property, we hereafter continue to refer to them simply as 'nitrogen sources' for simplicity. Our results show that the wild-type growth rate can be predicted from independent contributions of carbon and nitrogen sources in only 3 of our 28 conditions (Figure 2b). Significant interaction terms in all but three conditions signify the complex interdependencies throughout the metabolic network, thus underscoring the importance of testing each pair of sources systematically.

### Fitness determination of deletion mutants over the media conditions

In an effort to identify mutant growth defects specific to particular conditions, we derived a model designed to score growth rate for each deletion strain in a given condition relative to its growth under a reference condition (glucose:ammonium). First, the growth rate data (Additional file 1) were normalized for each experimental condition with respect to the glucose:ammonium reference (see Materials and methods). This controlled for the growth rate differences observable in wild-type cells across the different conditions (Figure 2; Additional file 2) and enabled us to focus on more subtle effects due only to the genetic perturbation. A modified z-score was then calculated for each mutant strain (see Materials and methods; Additional file 3). This measure is negative if the strain grew slower in the test condition than would be expected due to the nutrient environment alone, and positive if the strain grew faster than expected. The distribution of growth rates in the 701 wild-type replicates was used to assess the statistical significance of mutant effects in each condition and estimate a false discovery rate (FDR) for any gene-environment interactions (see Materials and methods; Additional file 4). Table 1 shows the number of deletions that grew slower or faster than expected at an FDR threshold of 20% (see Additional file 3 for a complete list of z-scores). While the large number of wild-type replicates allowed for confidence in the small differences in reference strain growth between various nitrogen sources when paired with glycerol, the mutant data on glycerol proved to be too noisy due to extremely slow growth to call mutant effects. Therefore, no growth rate (z-score) data are presented for mutant strains on glycerol.

### Observations in galactose concur with previous auxotrophic studies

**Figure 3:**
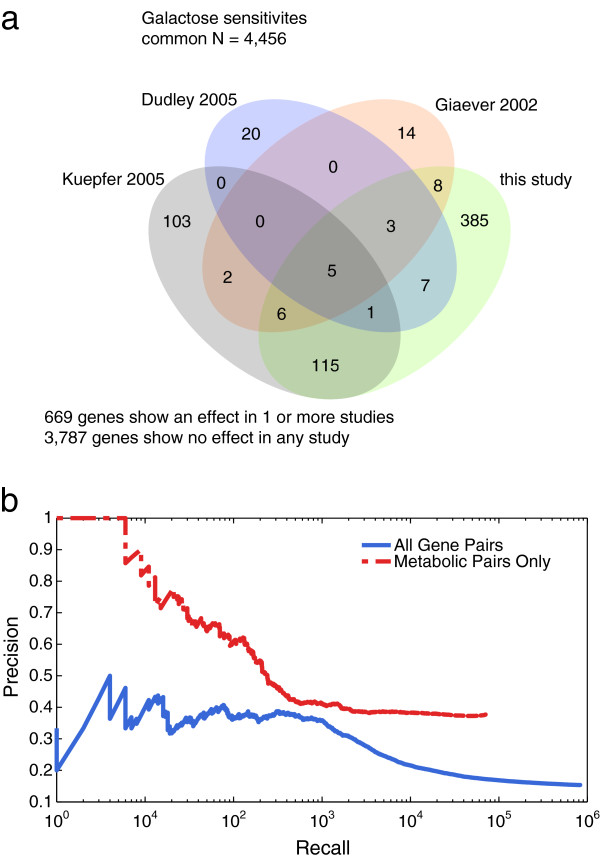
**Overlap with known galactose sensitivities and prediction of known functional associations. (a)** Overlap between mutants sensitive on galactose from several different studies. For this study, galactose sensitivity is defined as a significant z-score in four or more of our seven galactose conditions. N denotes the total number of genes the studies have in common. **(b)** Precision-recall analysis assessing the ability of gene-gene similarity to predict co-annotation to specific terms in the Gene Ontology. Results for all gene pairs are shown in blue, and results for a subset of metabolism-related genes (included in iMM904 model) are shown in red.

To build additional confidence in our high-throughput dataset, we compared lists of mutants deficient for growth under galactose to data from several previous studies that had tested the auxotrophic deletion collection in a variety of experimental conditions. Giaever *et al*. [1], Kuepfer *et al*. [13], and Dudley *et al*. [7] each included a condition in which galactose is the major source of carbon, and the overlap between the deletions that we call as effects in our galactose conditions and sensitivities collected from these three experiments is highly significant (Figure 3a; Additional file 5). We define a galactose-sensitive gene for this purpose as having a significant fitness defect in at least four of our seven galactose conditions and we obtain a list of 565 such genes (using FDR 20%; Additional files 3 and 4). This list covers approximately 50% of the sensitive genes identified in each of the three previous auxotrophic screens (Giaever n = 23, *P* < 10^−11^; Kuepfer n = 120, *P* < 2 × 10^−16^; Dudley n = 16, *P* < 10^−6^; hypergeometric; Figure 3a; Additional file 5). Additionally, we discover 385 mutants sensitive under galactose not revealed in any of these previous studies. For comparison, the overlap between two of the previous genome-wide studies (Giaever *et al.* and Kuepfer *et al.*) was only 15 genes, 12 of which are recovered in this study (Figure 3a). We suggest two primary reasons for the increased number of galactose-sensitive mutants discovered in our study. The first is that 47% of these new galactose-sensitive genes did not have a phenotype when only one nitrogen source (ammonium) was used. Thus, the testing of a wide-range of nitrogen sources revealed additional galactose-sensitive mutants. The second reason is that previous studies used more stringent thresholds for galactose phenotypes. Smaller quantitative measurements of fitness defects across multiple galactose:nitrogen source combinations allow for increased sensitivity in detecting galactose phenotypes compared with other studies.

Another possible explanation for differences between our galactose results and those from the Dudley *et al*. study is the absence of antimycin A in our media. Antimycin A inhibits energy production from respiratory pathways and forces the strains to ferment galactose. In our experiments, yeast had access to oxygen and could perform both respiration and fermentation with galactose as a carbon source, which is the natural metabolism of galactose by *S. cerevisiae*[14].

### Liquid culture validation of mutant fitness measurements

We independently validated our single mutant fitness measurements by measuring the growth rate of 40 mutants in a liquid growth assay performed across 20 of the experimental conditions (excluding ribose:arginine and all glycerol pairings; see Materials and methods). The overall correlation between wild-type strain growth rates from these two different approaches was 0.65 (*P* < 0.003; Pearson), suggesting general agreement between growth rates determined on solid and liquid media. We then adjusted the liquid growth scores, controlling for the wild-type rate in the given condition and the relevant mutant rate in glucose:ammonium so they would reflect condition-specific effects, similar to our modified z-score derived from the agar experiment. The Spearman rank correlation between the adjusted liquid growth score and our agar z-score (for 40 mutant strains × 19 conditions) was 0.34 (*P* < 2.2 × 10^−16^). Further excluding glucose conditions (which are generally sparser in the z-score data as a consequence of our use of glucose:ammonium as a reference) increases this correlation to 0.38. Thus, we conclude that there is reasonable agreement between the high-throughput measures and a lower-throughput liquid growth assay, including for condition-specific effects.

### Number of environmental sensitivities is correlated with single mutant fitness and genetic interaction degree

We compared our growth measurements with other quantitative phenotypes measured on the auxotrophic deletion collection. For example, genetic interaction mapping efforts have measured the single mutant fitness of all deletion strains from the auxotrophic background on synthetic complete media [3,15] and found a correlation between the magnitude of the fitness defect and the number of genetic interactions for each single mutant (genetic interaction degree). The prevailing explanation for this correlation is that genes that display a fitness defect represent the subset that are playing an active role under the condition tested, are additionally not completely buffered by other genes, and/or contribute to a wider variety of cellular processes. We observe a similar correlation between the single mutant fitness defect (as previously measured on synthetic complete media [3]) and the number of significant condition-specific sensitivities in our study (r = 0.33, *P* < 5 × 10^−100^; Pearson). Additionally, there is a partial correlation between the number of genetic interactions a gene has and the number of environments with which it interacts, even after controlling for single mutant fitness defect (r = 0.18, *P* < 5 × 10^−31^; Pearson). This echoes a previously observed correlation between genetic interaction degree and sensitivities in more complex chemical environments (r = 0.4, *P* < 10^−5^) [6,15]. These results confirm that our study is uncovering more effects for genes known to be pleiotropic or central under a variety of environmental backgrounds [7]. These findings also suggest that hubs are conserved across different network types, with many of the same genes conferring robustness to genetic, chemical, and environmental perturbations.

### Mutant sensitivity profiles are predictive of gene function

Previous genetic interaction studies have shown that high profile similarity for mutant sensitivity across varied environmental conditions or diverse genetic backgrounds (for example, genetic interaction profiles) is highly predictive of similar gene function [5,7,15]. We applied an analogous logic to our data to see if similar environmental sensitivity profiles would also be predictive of similar function. Using co-annotation to an informative set of Gene Ontology (GO) terms [16,17] as our standard for functional similarity, we ranked all pairs of genes by their profile similarity (Pearson) and evaluated these rankings with respect to known functional relationships. We measured a precision of approximately 35% at a recall of 1,000 gene pairs (2-fold over a random baseline of 17%; Figure 3b). Additionally, when we restrict our predictions to those genes with a known involvement in metabolism (663) we see a much higher precision (precision ~ 65% at recall = 100), though a similar performance over the increased background rate (1.7-fold over 38%; see Materials and methods). The higher performance for metabolism-related predictions is likely due to the direct relevance of the environmental conditions chosen to the study of basic metabolism. Thus, we have demonstrated an ability to predict general gene function using the guilt-by-association principle, and the diverse environments chosen for this assay are well-suited to reveal sensitivities in the metabolic network of this newly created prototrophic collection.

### Metabolic network models show modest ability to predict experimental data

**Figure 4:**
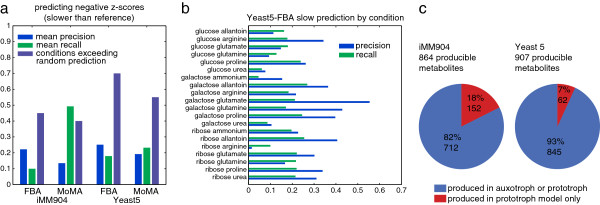
**Agreement between constraint-based modeling predictions and experimental observations. (a)** Assessment of constraint based modeling predictions for slow growth. Precision and recall (blue and green) were calculated for each model in each of 20 conditions (glycerol:* and glucose:ammonium excluded; Additional file 6) and means are shown here. The fraction of conditions in which predicted model mutants overlap significantly with significant z-score effects is shown in purple. **(b)** Precision and recall scores (as in **(a)**) for each individual condition using the Yeast5 model and standard FBA. **(c)** Number of producible metabolites for iMM905 and Yeast5 metabolic models. For each model the total number of producible metabolites was counted based on simulation in glucose:ammonium (see Materials and methods). The procedure was repeated for a model in which reactions involving auxotrophic marker genes (*HIS3*, *URA3*, *LEU2*, and *MET15*) were disabled. The chart shows the proportion of metabolites that the auxotrophic model fails to produce in red.

The prototroph growth data on minimal media presented here are uniquely suited to bring experimental data to bear on theoretical predictions of constraint-based analysis of metabolic networks. Constraint-based modeling is a widely used approach to study the metabolic capacity of genome-scale biochemical networks in steady state without requiring detailed enzyme kinetic parameters [8]. FBA is the most popular constraint-based approach to computationally predict the phenotypes under environmental and genetic perturbations and has been shown to successfully predict gene essentiality, and to a lesser extent, condition-specific essential status in yeast [13,18]. We used our sensitivity data to evaluate the ability of constraint-based models to predict subtler quantitative sensitivities in a condition-specific manner. We predicted biomass yield, a proxy for growth, in all conditions using two versions of the yeast metabolic network reconstruction: the more recent Sourceforge Yeast Consensus Reconstruction v5.35 (hereafter Yeast5) [19], and iMM904 [20]. Additionally, we applied two alternative algorithms to predict mutant phenotypes, namely standard FBA [21] and minimization of metabolic adjustment (MoMA) [22]. Predicted biomass production fluxes were normalized with respect to every mutant's predicted biomass production in glucose:ammonium and the wild-type prediction in each condition to make scores analogous to our experimental z-scores. The prediction of z-scores as opposed to raw growth rates was chosen to assess the adaptability of each model's performance in the face of varied environments, an admittedly more difficult scenario than predicting global or condition-specific essentiality. Though the output of the models is quantitative, many conditions predict only a few discrete levels of resulting biomass production and therefore yield identical predictions for the majority of mutants. The mode of the output accounted for between 39% and 95% of the predictions, so we assessed model performance by comparing the predicted set of slow mutants (below the mode biomass production) to our set of significant z-scores in each condition. Three metrics were collected to assess the performance of each model-method combination: average precision (across all 20 predicted conditions), average recall, and the number of conditions in which precision exceeded random expectation (at *P* < 0.05 hypergeometric; Figure 4; Additional file 6). Results for positive z-score prediction (above the mode biomass) are also available in Additional file 6 (see Materials and methods).

Prediction of condition-specific slow growth proved consistently above random expectation (Figure 4), though values of precision are much lower than those previously reported in predicting qualitative essentiality (>90% [18]). One key difference between our study and Snitkin *et al*. [18] (as with Dudley *et al*. in the section on galactose sensitivity above) is the latter's inclusion of antimycin A in the media, which inhibits energy production from respiration, whereas our strains could naturally respire and ferment. Our results show an advantage for the more recent Yeast5 model over the iMM904 model, as well as a slight advantage for standard FBA over MoMA. The Yeast5 model was able to perform above random expectation in 14 out of 20 conditions with a mean precision of 25% and a mean recall of 18% (Figure 4; Additional file 6). Recall scores for MoMA were generally higher than for FBA owing to a much smaller fraction of the predictions equal to the mode, though this was generally associated with a loss of precision. Galactose conditions appear to be well captured by the two models, and consistently perform above random. By contrast, all three conditions for which no model-method achieved significance involved glucose (glucose:allantoin, glucose:glutamine, glucose:urea). Thus, while the overall performance demonstrates an above-random ability of these models to predict quantitative and condition specific perturbation effects, their modest precision and recall scores (<50%) suggest substantial room for improvement.

An examination of false positives (predicted sensitive by the model but not observed in the data) and false negatives (observed sensitive, not predicted) showed some functional coherency. Specifically, Kyoto Encyclopedia of Genes and Genomes (KEGG) enrichment of false positives in many conditions revealed connections to central carbon metabolism (for example, the tricarboxylic acid cycle), and half of the conditions showed enrichment for the KEGG sulfur metabolism pathway in the model for false positives (Additional file 6). This suggests potential pathways that may need attention for the development of improved models.

We also attempted to leverage existing metabolic models to demonstrate the widespread metabolic consequences of these common auxotrophies. To accomplish this, we ran the models again using prototrophic and auxotrophic versions of the network on glucose:ammonium and characterized each metabolite as either: i) produced in the auxotroph and the prototroph; ii) produced in the prototroph only; or iii) included in the model but not produced in an optimal solution (see Materials and methods). The simulations show that a significant proportion of producible metabolites (18% in iMM904 and 7% in Yeast5; Figure 4c) are unavailable in the auxotrophic network. This means that consequences of using auxotrophic strains, even under supplementation for their specific deficiencies, may have a broader impact than expected. It is our hope that the collection and accompanying growth data presented here will prove invaluable to the metabolic modeling community as it continues to refine the structure of its models as well as their underlying biological assumptions.

### Broad environmental surveys address incomplete gene annotations

A primary motivation for measuring fitness across diverse environments is the discovery of novel phenotypes for mutants that have near wild-type fitness under previously tested conditions. The existence of such mutants in a eukaryotic genome with approximately 6,000 genes is driven by two main factors. The first is genetic redundancy, whereby genes are performing vital functions within the cell, but their importance is not captured by single mutant phenotypes because other genes are present that buffer the loss of function. This occurs at both the level of individual genes buffering one another (for example, duplicate genes [23,24]) and the level of larger network structures (for example, parallel pathways). These buffered functions are rapidly being mapped by genetic interaction studies that delete multiple genes simultaneously [2,4,11,15,25,26]. The remaining contributing factor is environmental robustness, whereby a gene presumably has an important function under some evolutionarily relevant circumstance that is not reflected in a laboratory environment (for example, nutrients/media, temperature, stress). Thus, an important motivation for complete pairwise coverage of basic metabolic conditions is the detection of novel fitness defects for genes that become necessary only as the condition space is more broadly surveyed. Interestingly, of the 729 remaining uncharacterized mutants in the auxotrophic collection for which we have single mutant fitness measurements in synthetic complete media, a significant fraction of them (609) have a fitness greater than 99% of wild-type (hypergeometric *P* < 7 × 10^−66^) [27]. Despite the ever-increasing availability of high-throughput genomic data for these genes, the task of eliminating this set has seen only marginal success since 2007 [28]. It is possible that these genes (many of which only have orthologs in other yeasts) may be responsible for functions needed in the native environment of yeast but unnecessary under standard laboratory conditions. Still others may be required in the lab, but only after varying the nutrient conditions. The focus of recent chemical genomics work on subjecting yeast to an extremely broad range of chemical environments is helping to address these genes [5,6], but auxotrophy in the deletion collection had precluded measurements of growth on simple but directly relevant metabolic conditions. Here we address the potential impact of these data on both uncharacterized genes and genes of little phenotypic consequence in standard conditions.

### Novel effects for genes with high fitness in standard conditions

**Figure 5:**
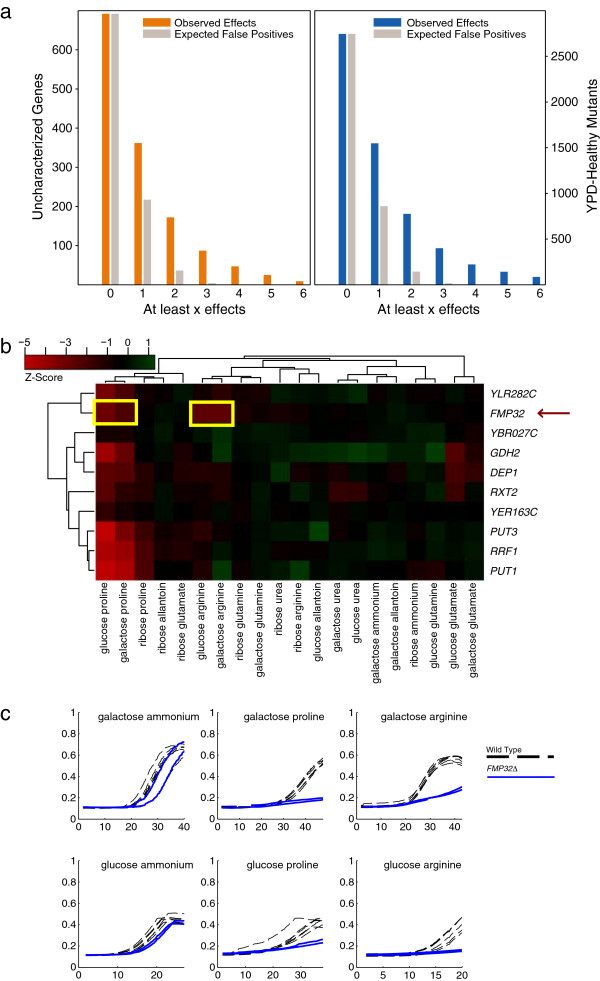
**Measuring effects for poorly characterized genes. (a)** Counting slow-growth effects for under-characterized genes. Histograms show the total number of mutants with at least x significant slow-growth effects in our data from the set of uncharacterized genes (left, orange), and genes with little to no fitness defect on synthetic complete media (right, blue; single mutant fitness > 98% of wild-type). As a control, the expected number of false positives (20% of significant effects in each condition) were randomly distributed among all genes, and the number of effects for each gene was counted again. Gray bars show the mean of 1,000 such randomizations. **(b)** Z-score data show specific growth defects for the uncharacterized gene *FMP32* when grown on proline or arginine. **(c)** Liquid growth confirmations for effects highlighted in X-axis, time in hours. Y-axis, optical density. **(b)**. Two replicates of *FMP32* mutants are shown (blue line) along with six replicates of a wild-type strain (black dashed line) in two proline and two arginine conditions. The effects are pronounced when compared to observations in similar ammonium conditions.

As described earlier, we observed that the number of significant effects in our data can be weakly predicted by single mutant fitness in synthetic complete media. However, nearly 40% of the *S. cerevisiae* genome shows little to no such effect. Of the genes in this study with single mutant fitness scores greater than 99% of wild-type under synthetic complete media, more than 50% of them (1,548/2,745; Figure 5a) show at least one significant slow-growth effect outside of glucose:ammonium. Multiple random assignments of the number of expected false positives (20% of effect counts listed in Table 1) demonstrate that only approximately 30% of genes should show an effect. Additionally, 5% (142/2,745) show significant effects in five or more distinct non-glucose:ammonium conditions compared to a random expectation of 2.6 × 10^−5^ (<<1/2,745). For example, *prs2Δ0* (the *PRS2* gene encodes one of the four phosphoribosyl-pyrophosphate (PRPP) synthetases encoded in the genome; these synthetases are required for nucleotide, histidine, and tryptophan biosynthesis) has a single mutant fitness of 1.02 in synthetic complete media [3] but shows significant growth defects in 14 different conditions These conditions are highly coherent, including all seven galactose conditions, all ribose conditions (except ribose:arginine) and no conditions involving glucose except glucose:proline. *PRS2* is highly expressed under fermentative conditions [29]. Another example is *ICL1*, which facilitates a key reaction of the glyoxylate cycle, and shows slow growth effects in nine (non-glucose:ammonium) conditions despite a single mutant fitness score slightly greater than that of wild-type under standard lab conditions (1.03) [3].

### Novel phenotypes for uncharacterized ORFs

Approximately 13% of the *S. cerevisiae* deletion collection is composed of uncharacterized ORFs [27], 692 of which are included in this study. Nearly 25% of these uncharacterized genes show a significant effect in two or more non-glucose:ammonia conditions (172/692; Figure 5a) compared to the 4% expected given our FDR.

One such example with a very specific nitrogen sensitivity signature is *FMP32*. The *fmp32Δ0* strain displays dramatically decreased fitness under arginine and proline conditions. While the protein product of *FMP32* has been detected in highly purified mitochondria [30], the gene is otherwise uncharacterized. The *fmp32Δ0* strain was included in our liquid confirmation assay and these sensitivities were confirmed in this independent, small-scale assay (Figure 5b). This highly specific signature appears to be completely unique to the *fmp32Δ0* strain, as no other mutant in the collection shows a similar sensitivity profile.

The genes with the highest profile similarity to *FMP32* are *PUT1*, *PUT3*, and *RRF1*, which have been previously implicated in proline utilization (*PUT1*, *PUT3*[31]) and mitochondrial ribosome recycling/mitochondrial protein synthesis during respiration (*RRF1*[32,33]). *PUT3* induces *PUT1* transcription when proline is present as the best available nitrogen source and the latter (along with *PUT2*) is responsible for the conversion of proline into glutamate for further use as a nitrogen source. Our analysis suggests that *FMP32* is similarly involved in the respiratory response under proline, though the reason for its additional sensitivity under arginine remains unclear. These examples show the utility of interactions between genes and simple environments in uncovering the function of both individual uncharacterized genes and genes without a previously observed fitness defect in more complete media.

### Clustering of metabolic conditions reveals carbon source as primary factor driving mutant profiles

**Figure 6:**
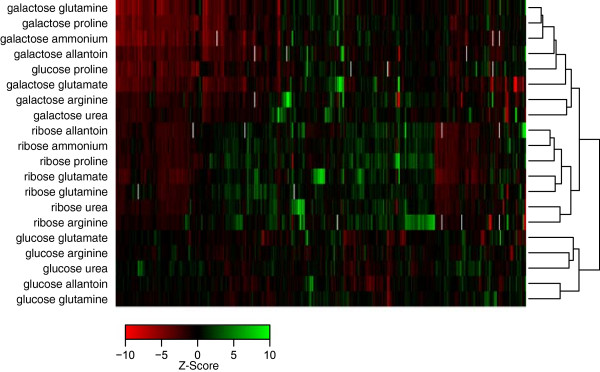
**A clustergram of Z-scores for the 500 mutants with the highest variance.** The data have been hierarchically clustered in both dimensions. Conditions organize themselves primarily by carbon source, falling into three distinct clusters.

Just as gene-gene correlation predicts functional similarities, we expect a high correlation between condition pairs to reflect a substantial overlap in the cellular machinery required to utilize the provided carbon and nitrogen sources. When our matrix of z-scores is hierarchically clustered in both the gene and condition dimensions, a structure clearly driven by carbon sources emerges (Figure 6; see Materials and methods). All of the glucose conditions cluster together, as do both the galactose and ribose conditions. The sole exception to this is glucose:proline, which falls in the galactose cluster. We attribute this observation to the fact that the utilization of proline as a nitrogen source requires some respiration. The glucose:proline signature reveals sensitivity in a number of respiratory deficient mutants, which is atypical for glucose conditions in general since fermentation is generally preferred over respiration when cells are grown on glucose. This respiration-dependent signature is strong enough to place the glucose:proline profile in the galactose cluster where one would expect a modest profile contribution from both respiration- and fermentation-related processes (Figure 6), as is observed in growth on galactose [14].

### Matrix factorization distinguishes carbon from nitrogen effects

Further examination of gene and environmental profiles after clustering revealed cases where a gene (for example, *FMP32*) exhibited an effect in multiple instances of a particular nitrogen source (for example, proline or arginine), but without a specific pattern with regard to carbon source (or vice versa). This is expected behavior for genes required for the utilization of a particular carbon/nitrogen source regardless of the context. In order to more formally extract a list of sensitivities for each source of carbon or nitrogen regardless of its partner, we employed a method known as non-negative matrix factorization (NMF) [34,35] to decompose our experimental data into a collection of characteristic source signatures. When a matrix of these source signatures is multiplied by a matrix describing the source composition in each of our conditions, the result should approximate our experimental observations. NMF allows us to run this multiplication in reverse and fit the source signatures as an unknown factor. These source signatures are available in Additional file 7, and several of them demonstrate enrichment for related GO terms and KEGG pathways.

One example of a decomposed signature involves genes that are sensitive when glutamate is chosen as a nitrogen source. These genes are enriched for annotations relating to endocytosis, endosome and vacuole related transport, and retrograde transport (Additional file 7). Extracellular glutamate decreases cellular amino acid permease activity by redirecting intracellular trafficking of the permease Gap1 from the plasma membrane to the vacuolar membrane [36]. Many of the mutations in our glutamate signature increase Gap1 activity by misdirecting the protein to the plasma membrane [37]. Although *GAP1* is transcribed at equal levels in cells grown on urea and glutamate, permease activity in urea grown cells is 100 times higher than glutamate-grown cells [38]. Inappropriate Gap1 activity is toxic in the context of high concentrations of single amino acids [39], and we speculate that the inappropriate trafficking in these mutants causes high levels of permease activity that inhibit cell growth.

Many mutants (92) appear in both the galactose and ribose signatures, and overlapping GO enrichments in these conditions reveal many of these genes to have known involvement in various aspects of respiration. For example, enrichment for GO terms relating to mitochondrial organization and translation, as well as 'aerobic respiration', appear highly significant in both of these signatures (Additional file 7). Exceptions include GAL pathway mutants that fall uniquely into the galactose carbon signature ('galactose metabolic process' *P* < 1.3 × 10^−4^) and genes involved in acetyl-CoA biosynthesis that appear to be specifically sensitive under ribose (*P* < 1.4 × 10^−6^). As more complex environments are mapped, multivariate statistical techniques will become increasingly important in determining which environmental constituents are actually relevant to which experimental observations, and care should be taken when designing experiments to ensure their successful application (for example, complete combinatorial coverage of relevant environmental factors).

### Environmental and genetic perturbations can provoke similar cellular states

**Figure 7:**
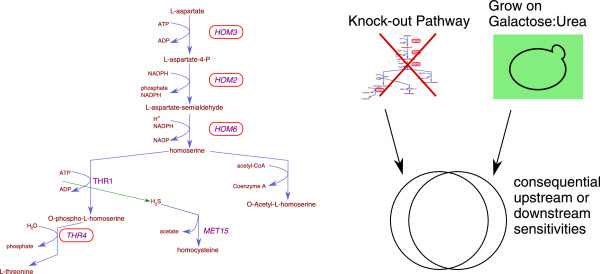
**Comparison of sensitivity profiles from environmental to genetic perturbations.** High dimensional sensitivity information for mutants in threonine biosynthetic pathway (circled in red) were obtained from SGA experiments [15]. These profiles correlate with the sensitivity profile obtained in this study when strains are grown on galactose:urea. This suggests a correspondence between the internal states of the cells when grown in a specific environment, and when subjected to a specific genetic perturbation. For example, *hom2Δ*, *hom3Δ*, *hom6Δ*, and *thr4Δ* mutants would all be expected to accumulate aspartate because these mutants shut down a major metabolic shunt for aspartate. The phenotypic similarity in genetic interaction space between these mutants and growth on galactose:urea suggests that growth on galactose:urea may cause the internal accumulation of aspartate or some other metabolic intermediate unique to the *hom2Δ*, *hom3Δ*, *hom6Δ*, and *thr4Δ* mutants*.*

Beginning to test the immense space of possible environmental and chemical conditions combined with experiments that have queried the space of genetic perturbations [15] allows us to investigate how these spaces interrelate. For example, if mappings can be found between them, we can apply knowledge from the already extensively mapped genetic perturbation networks to the intractable space of environmental variation. While the sensitivity profile for a given condition most certainly includes genes directly required for the processing of the provided raw materials (for example, the galactose metabolism pathway under galactose conditions), it also contains information about genes that, though not directly involved, are nonetheless indirectly required for optimal cell growth. These profiles then reveal much more than the functions of genes for which we measure a fitness defect, and in fact give us a high dimensional fingerprint of the internal cellular state. We propose that genetic perturbations may put the cell into a very similar state as would an alteration of the environment. For example, the deletion of a gene that encodes a transporter may exhibit a profile that mimics the wild-type profile in an environment where the corresponding substrate is absent. Downstream consequences of the environment or genetic perturbation may cause subtle and seemingly unexpected sensitivities. Thus, genetic perturbation experiments and environmental perturbation experiments may both result in the same phenotypic profile. A similar principle has been demonstrated through the observation that deletion mutants with similar double mutant sensitivity profiles tend to be functionally related [15]. Parsons *et al.*[5] first applied this principle to predict drug targets, reasoning that a genetic sensitivity profile on a chemical that targets an individual gene would be similar to a sensitivity profile of a strain with the corresponding gene deleted. When we compared sensitivity profiles from our condition experiments to that of query-deletions crossed into the auxotrophic deletion collection via SGA [15], we found several interesting cases where genetic perturbation profiles significantly overlapped with sensitivity profiles from our environmental perturbations (see Materials and methods). For example, the queries in the top 10% in terms of similarity to galactose:urea are enriched for members of the threonine and methionine biosynthesis pathway (*hom2*, *hom3*, *hom6*, *thr4*; Figure 7; GO:0006566 'threonine metabolic process' *P* < 4.5 × 10^−2^; KEGG 'glycine, serine and threonine metabolism' *P* < 2.9 × 10^−2^). The strength and specificity of this similarity is not driven by a handful of mutants in the collection, but instead by trends across a much larger set of genes. We speculate that the profile similarity in this case may be due to accumulation of aspartate, which is upstream of homoserine and threonine biosynthesis, and is excreted in part through urea production. Growth on urea in the setting of the respiratory growth of galactose may result in the accumulation of aspartate.

The idea of comparing environmental and genetic perturbations can be generalized to other genome-wide perturbation data as well. For example, we observe significant correlations between our glutamate signature and a rapamycin sensitivity profile as measured by two different chemical genomic screens (Hillenmyer *et al. P* < 10^−18^[6]; Parsons *et al. P* < 10^−9^[5]). The enrichment for transport-related terms observed in the glutamate signature (above), and its similarity to a rapamycin profile make sense given that rapamycin redirects trafficking of Gap1 from the plasma membrane to the vacuole [40]. Thus, the same set of mutations in vesicle trafficking that lead to inappropriate expression of Gap1 permease activity in cells grown on glutamate also cause inappropriate permease activity following rapamycin treatment.

## Conclusion

The creation of the original yeast deletion collection has had a profound impact on the way in which reverse genetic experiments are performed. Yet despite a staggering number of successful studies, the inherent auxotrophies create a major blind-spot in a fundamental area of cellular function, and previous reviews of the topic have called for the creation and use of standardized prototrophic strains for metabolic experiments [9]. Recently, Mülleder and colleagues [41] have addressed the deletion collection auxotrophies by introducing a plasmid containing sequences for *HIS3*, *URA3*, *LEU2*, and *MET15.* The resource used in this study differs in that *URA3*, *LEU2*, and *MET15* are in their native genomic locations, with the exception of *HIS3*, which is provided by *Schizosaccharomyces pombe HIS5* under the SGA reporter [11]. Without the necessity for plasmid selection, or possible effects on gene expression due to non-chromosomal location, we anticipate that our deletion collection will see frequent use by experimentalists.

The use of a genome-wide prototrophic strain collection enables truly informative sensitivity screening in metabolically controlled conditions. This represents a first step in probing how nutrients in the environment jointly affect cellular response with or without additional genetic perturbation. This study demonstrates that much work is yet to be done to understand growth in even simple environments. A solid grasp of the surprisingly complex responses to simple environments will add much needed context to studies done in more complex environments.

This study has demonstrated the potential of this collection, when screened against simple environments, to uncover phenotypes for hundreds of mutants that are phenotypically normal in standard lab conditions. We believe that the stock of simple experiments that might reveal a phenotype for these mutants has not yet been exhausted and expect that this whole-genome prototrophic collection will be an invaluable resource to the community. The rising number of metabolomics studies, fueled in part by the increasing accuracy of experimental mass-spectrometry, as well as the growing interest in metabolism as central to many common ailments in humans, make it more important than ever to properly design metabolically relevant experiments in the model eukaryote *S. cerevisiae*. Central to that goal is a version of the deletion collection that is unhindered by historical auxotrophic requirements.

For example, while central metabolism is unrivaled among cellular processes with respect to our ability to make *in silico* predictions from constraint-based metabolic models, it is far from a fully understood system. Our results show a generally weak ability to predict condition-specific sensitivities, though performance is clearly above a random baseline. The prediction of condition-specific sensitivities is admittedly more difficult than the prediction of sensitivities in general, but it was our estimation that FBA and MoMA would be well suited to approximate our observations given our simple experimental setup. Their only moderate success in doing so demonstrates the current limitations of constraint-based modeling and the difficulty of relating models built from biomass predictions to quantitative growth rate data. There might be several possible reasons for the discrepancy between *in silico* and *in vivo* results. First, the success of predicting growth defects hinges on the proper formulation of biomass composition. While a single biomass composition is used for all our simulations, it likely changes across environmental conditions. Future studies could address this issue by measuring the composition of yeast cells under different nutrient settings. A second limitation of purely flux-based models is their inability to make predictions about components that have an indirect effect on metabolism. Consider, for example, the enrichment for transport-related genes whose deletion confers glutamate-specific sensitivities. Their putative role in nutrient sensing and signaling reflects the fact that, despite its constrained nature, the metabolic network operates as part of a much larger and more dynamic network. More generally, the basic constraint-based modeling approaches ignore regulatory mechanisms. Several attempts have been made to bridge this gap and they rely either on 'omic' data to constrain the activity of specific reactions [42-44] or on integrating a mathematical representation of gene regulation with the metabolic model [45-47]. We feel that the availability of this whole-genome collection and accompanying growth data well suited to studies of metabolism will help the community to develop and test novel models and methods to better capture the operation of the greater cellular network.

Central to the understanding of the network as a whole is the idea that a whole-genome screen reveals indirect as well as direct consequences of the perturbation tested. Positive gene-environment interactions under ribose conditions may well illustrate this point. The median z-score for the 166 genes annotated to 'chromosome segregation' in GO is negative for all seven galactose conditions, yet positive for all seven ribose conditions (binomial sign-test *P* < 6.2 × 10^−5^). We believe this shift may be explained by fundamental cellular rate limitations. Failure to segregate chromosomes in the midst of even moderate growth (for example, galactose) can have very severe consequences, ultimately limiting growth rate, whereas comparatively slow growth (for example, ribose) affords additional time for slowly segregating mutants to complete segregation. These mutants grow faster than we expect despite no apparent link between carbon metabolism and chromosome segregation. Thus, growth rates under one condition disclose information about the interplay between a wide variety of cellular subsystems, giving us a readout of the internal cellular state. Similarly, a mutant profile across many environments gives us information about how essential that gene may be in any of those various cellular states, in addition to elucidating any direct role that gene may have in direct utilization of the provided nutrients. Analysis of our growth data recapitulated the role of vesicle trafficking in the regulation of the amino acid permease Gap1, relating growth on glutamate to the drug rapamycin. This broader view of whole-genome screen information then allows for integration of profiles across different perturbation types (chemical, genetic, environmental), and should ultimately aid us in applying knowledge gained in one arena to observations made in another.

## Materials and methods

### Construction of a prototrophic deletion collection

As recently described [48], the strains in the standard *MAT****a*** deletion collection (*MAT****a ****yfgΔ0::KanMX his3Δ1 leu2Δ0 met15Δ0 ura3Δ0*) [1] were mated to a *MATα can1Δ::STE2pr-SpHIS5 his3Δ1 lyp1Δ0* strain, creating diploids (selection on minimal media + his + G418). These were sporulated and successive pinnings on selective media were used to select prototrophic *MAT****a*** strains carrying each deletion allele. These prototrophic strains were organized into an array of 16 plates including one entire plate of the wild-type strain (*hoΔ::KanMX*), with additional wild-type replicates in each row and column of every plate (701 in all). The entire prototrophic collection is available upon request, as is the individual SGA-ready prototroph strain for crossing into other collections.

### Media preparation

Minimal growth media were prepared using yeast nitrogen base (BD Difco, Sparks, Maryland,) with the specified carbon and nitrogen sources. Carbon sources included glucose, galactose, ribose, and glycerol. Nitrogen sources included ammonium, allantoin, arginine, glutamate, glutamine, proline, and urea. Carbon sources were provided at a concentration of 2%; nitrogen sources were 3.8 mM with respect to nitrogen.

### Calculation of growth rate

Sixteen 16 × 24 well plates were grown in 28 chemical conditions for 24 to 48 hours. Plates were scanned on a flatbed transparency scanner at 0, 5, 10, 24 and, in the case of glycerol, 48 hours. Each condition is composed of one carbon source and one nitrogen source. In total, 4,772 mutants were grown, and colony areas were extracted from tiff images by CellProfiler [49] and precise time points were taken from EXIF data in the digital images. These values were used to compute an estimate of the growth rate of each colony equal to the slope of the least-squares linear fit of area (pixels) to time (seconds). Colonies with insufficient data were given a growth rate of NaN, colonies with a negative calculated growth rate were defined to have a growth rate of 0.

### Definition and construction of a reference condition

Six replicates of the glucose:ammonium combination were merged to form a reference condition, establishing a baseline score for each deletion. The six replicates were first normalized to each other to control for differences in the overall scale of growth rates, then averaged together according to the following procedure. For each array plate (p) the glucose:ammonium replicate with the fewest missing data points was held out (Plate_A_) and the remaining five replicates were LOWESS smoothed (window size = 50% of available data) and normalized by:

GAplatep'=GAplatep×PlateAlowessGAplatep

The result of this approach is quite robust to the choice of Plate_A_, and so we used whichever replicate had the fewest number of missing values and would therefore provide the most complete LOWESS fit. After normalizing five replicates to the sixth, all six were averaged together to create one reference plate, and this procedure is repeated 16 times to create a glucose:ammonium reference for each array plate.

### Normalization of experimental rates against reference

In every experimental condition (Y), each plate was LOWESS smoothed (window size = 50% of available data) against the constructed glucose:ammonium reference plate, then normalized:

CondYplatep'=CondYplatep×GArefplowessCondYplatep

### Recovery of missing data

In certain cases, a growth rate of NaN was assigned to a colony due to insufficient data being collected by CellProfiler. In an effort to recover any good data, these cases were visually inspected by five researchers operating independently and a vote was taken to determine whether to leave it as missing data (NaN) or assign it a growth rate of 0, indicating that the colony appeared to be correctly plated but non-viable. In total, 1,362 of 2,601 colonies were recovered this way.

### Transformation from normalized rates to z-scores

For each array plate, at each position, a strain-wise standard deviation is calculated across the residuals of the six glucose:ammonium (GA) replicates.

Similarly, a plate-wise standard deviation is calculated that accounts for the general growth variation on the plate, separately for each condition. These are then combined, and a z-score measure is calculated for each strain on each experimental plate:

z=CondYplatep'−GArefpstddevstrain2+stddevplate2

These z-scores are an expression of the difference in magnitude and direction between the growth observed at each position of a plate under a given condition from the same position (and hence deletion) under the reference GA model.

### Spatial smoothing procedure

The plate level spatial smoothing filter is similar to that found in [3]. First, temporarily replace any extreme values (top and bottom 5%) along with NaNs with the plate mean. Second, replace previous NaN positions with values from a two-dimensional symmetric gaussian filter. Third, compute and subtract the residual between the two-dimensional smoothed plate and its mean.

### Choosing effect thresholds

Each condition had 701 wild-type replicates. The mean and standard deviation of the set of wild-type z-scores were used to define a normal distribution against which *P*-values for the experimental z-scores could be calculated. This information allowed the use of Benjamini-Hochberg procedure to establish condition-specific effect thresholds as a function of a desired FDR (Additional file 4).

### Liquid growth confirmation assay

The growth rate of 40 mutants in a liquid growth assay was measured across 20 of the experimental conditions excluding ribose:arginine and all glycerol pairings. Liquid culture assays were not performed for the ribose:arginine conditions because the combination of these carbon and nitrogen sources did not allow arginine to maintain adequate solubility over the duration of the experiment. The precipitation of arginine prevented accurate optical density readings from being obtained and thus these data were excluded from our subsequent analyses. Six replicate wells contained the wild-type strain and each mutant strain was represented twice. Cells were pre-grown on glucose:ammonia medium and diluted at a low density into the growth medium of interest. Growth rates were determined as the maximum optical density (saturation) divided by the time to saturation. A simple model was favored in order to robustly accommodate drastic differences in curve characteristics between fast growth and slow growth conditions (for example, galactose versus ribose).

We adjusted the liquid growth scores by dividing the mean of mutant growth slopes by the mean of wild-type growth slopes in the relevant condition. We further normalized these scores by dividing them by the corresponding adjusted mutant score in glucose:ammonium so they would reflect condition-specific effects, similar to our modified z-score derived from the agar experiment.

### Gene Ontology and KEGG enrichments and co-annotation standards

GO and KEGG annotations were downloaded in January 2011 [16,50].

### Genome-scale metabolic modeling (FBA and MoMA)

Two *S. cerevisiae* metabolic models were used for mutant biomass prediction. The Yeast Consensus Reconstruction version 5.35 (Yeast5) [19] and iMM904 [20]. Yeast5 consisted of 898 ORFs, 2,031 reactions and 1,594 metabolites and the iMM904 model contained 901 ORFs, 1,597 reactions and 1,234 metabolites. Default biomass descriptions were used for both models.

Wild-type biomass production flux for each condition was obtained using FBA [21] in MATLAB with the COBRA Toolbox [51], which assumes optimal biomass production (that is, maximum biomass yield). Mutant biomass flux was predicted using both FBA [21] in MATLAB with the COBRA Toolbox [51] and MoMA [22] in MATLAB with the ILOG CPLEX optimization suite. MoMA was formulated as a quadratic programming problem, whereby mutant fluxes were selected that minimized the Euclidean distance from an optimal wild-type flux distribution. The yeast wild-type flux distribution was calculated as a network flux solution producing maximal biomass flux, determined by FBA, with minimal total fluxes [52].

FBA and MoMA biomass fluxes were correlated with both raw and normalized (z-score) experimental growth rates using the Spearman rank correlation. Predicted biomass fluxes were also normalized for comparison to experimental growth rate z-scores (separately in each condition):

$$\begin{array}{l} \mathrm{NormalizedFlu}x_{\mathrm{\Delta x}}\mathrm{Con}d_{Y}=\frac{\mathrm{RawFlu}x_{\mathrm{\Delta x}}\mathrm{Con}d_{Y}}{\mathrm{RawFlu}x_{\mathrm{\Delta x}}\mathrm{Con}d_{\mathrm{Glu}:\mathrm{Amm}}\times \mathrm{RawFlu}x_{\mathrm{wild}-\mathrm{type}}\mathrm{Con}d_{Y}} \end{array}$$

Prediction of positive z-scores was also carried out, though performance was generally below random expectation (Additional file 6). This is likely due to the fact that many positive z-scores corresponded to raw growth rates for mutants that were faster than wild-type under the same condition, a consequence that FBA- and MoMA-based methods would find difficult or impossible to predict.

To calculate the effect of gene deletions on the metabolic network (Figure 4c), sets of producible metabolites were calculated for the complete model, and for a mutant with all four auxotrophic marker genes deleted. Producible metabolites were calculated for both iMM904 and Yeast5 models in the glucose:ammonium media condition by adding a special exchange reaction for each metabolite and iteratively optimizing flux exported through that reaction. If the export flux for a given metabolite exceeded 0.001 (with an upper and lower bound on internal reactions set to ±1,000), it was classified as 'producible.' A non-zero threshold is required to limit false positives as a result of numerical errors. The threshold was determined to be robust by scaling the upper and lower bounds, as well as the threshold by a large constant and counting the number of producible metabolites. Obtaining consistent results in these experiments led us to conclude that numerical errors are an order of magnitude smaller than contributions from stoichiometry.

### Source signature decomposition via modified non-negative matrix factorization

Growth data were decomposed using a variant of NMF [34]. Following transformation to z-scores, the data were made binary using condition-specific FDR estimates as thresholds (20% FDR; Additional file 4). The resulting Boolean *Data* matrix was treated as numeric and served as the target for decomposition. Genes without any significant z-scores in any condition (empty rows) were removed, as were the columns involving growth on glycerol. We then defined a *Coefficient* matrix that related *Condition* rows in the data to their component *Sources.* This matrix then had C columns and S rows. For example, the glucose-urea column has a 1 in the glucose row and a 1 in the urea row. Our task is then to find a *Signatures* matrix (Genes × Sources) such that the difference between the *Data* matrix and the *Signatures-Coefficients* product is minimized:

$$\mathrm{Data}\left(G,C\right)\approx \mathrm{Signatures}\left(G,S\right)\times \mathrm{Coefficients}\left(S,C\right)$$

To ensure linear independence among the columns of the *Coefficient* matrix, we removed all but one glucose:ammonium (glucose:ammonium01) column, removing the same columns in the *Data* matrix. Traditional NMF would use a multiplicative update algorithm applied to both the *Signature* and the *Coefficient* matrix to find the best fit to the data; however, we chose to fix the *Coefficient* matrix at the initial defined values (0 or 1). This gives each *Signature* column equal weight and prevents over-fitting caused by the sparsity of the *Data* matrix and the dramatically different number of non-zero elements from one column to the next. The multiplicative update was applied for 20 iterations, though in practice the results converged in fewer than 10, and repeated trials from different random initializations of the *Signature* matrix showed the results to be quite stable. Genes were added to the signature list in Additional file 7 if their value exceeded 0.4.

### Comparison to SGA data

For the comparison to auxotrophic SGA data represented in Figure 7, the SGA data were taken from [15]. The SGA data and the z-score data (Additional file 3) were independently normalized so that row and column vectors had a euclidean length approximately equal to 1, and missing values were set to 0. Inner product was then used to measure the similarity between SGA 'queries' and environmental profiles. The top 10% of queries in each condition were checked for enrichment for GO terms and KEGG pathway annotations, and the resulting *P*-values were Bonferroni corrected to account for the number of terms/pathways tested against.

### Comparison with previous whole-genome screens on galactose

Figure 3a uses a publicly available image for the basis of the Venn diagram. This image is used with permission under the terms of the Creative Commons Attribution-Share Alike 3.0 Unported license [53].

## Abbreviations

FBA: flux balance analysis; FDR: false discovery rate; GO: Gene Ontology; KEGG: Kyoto Encyclopedia of Genes and Genomes; MoMA: minimization of metabolic adjustment; NMF: non-negative matrix factorization; ORF: open reading frame; SGA: synthetic genetic array.

## Competing interests

The authors declare that they have no competing interests.

## Authors’ contributions

DCH, OGT, CLM, and AAC conceived the study. DCH and AAC performed the laboratory experiments. CN provided experimental resources for the liquid growth assay. BJV, CP, and TS analyzed the raw data. BJV, CP, and EK designed and performed further computational analysis and experiments. BJV, DCH, BS, BP, CLM, and AAC wrote the manuscript. All authors read and approved the final manuscript.

## Supplementary Material

Additional file 1 Raw growth rates for all 28 conditions and all 4,772 strains. Click here for file

Additional file 2 Raw growth rates for 701 wild-type replicates in all conditions. Click here for file

Additional file 3 z-score data for 21 conditions. Click here for file

Additional file 4 FDR 10% and 20% thresholds for z-score data. Click here for file

Additional file 5 A list of 565 galactose-sensitive genes as well as overlap details between this set and three other full genome studies using the auxotrophic collection on galactose. Click here for file

Additional file 6 **Summary of fast/slow prediction accuracy of FBA models (sheets 1 and 2).** Also contains KEGG pathway enrichments for sets of genes predicted to be sensitive but not observed and vice versa (sheets 3 and 4). Click here for file

Additional file 7 List of genes in each signature and GO enrichments. Click here for file
